# The Utility of Pharmacogenetic-Guided Psychotropic Medication Selection for Pediatric Patients: A Retrospective Study

**DOI:** 10.3390/pediatric13030049

**Published:** 2021-07-28

**Authors:** Merlin Ariefdjohan, Yee Ming Lee, Danielle L. Stutzman, Sean LeNoue, Marianne Z. Wamboldt

**Affiliations:** 1 Department of Psychiatry, Child and Adolescent Mental Health Division, University of Colorado Anschutz Medical Campus, Aurora, CO 80045, USA; merlin.ariefdjohan@cuanschutz.edu; 2 Department of Clinical Pharmacy, Skaggs School of Pharmacy and Pharmaceutical Sciences, University of Colorado Anschutz Medical Campus, Aurora, CO 80045, USA; yeeming.lee@cuanschutz.edu (Y.M.L.); danielle.stutzman@childrenscolorado.org (D.L.S.); 3 Pediatric Mental Health Institute, Children’s Hospital Colorado, Aurora, CO 80045, USA; 4 Rogers Behavioral Health, Nashville, TN 37205, USA; sean.lenoue@rogersbh.org; 5 Helen and Arthur E. Johnson Depression Center, University of Colorado Anschutz Medical Campus, Aurora, CO 80045, USA

**Keywords:** pharmacogenetic testing, psychiatric disorders, CPIC/FDA, practice recommendations

## Abstract

Background: To describe trends and clinical experiences in applying commercial pharmacogenetic testing among pediatric patients with neuropsychiatric disorders. Methods: Demographic and clinical data of patients receiving GeneSight^®^ testing from January 2015 to November 2016 at an urban pediatric hospital were retrospectively extracted from medical charts. Outcome data included pharmacogenetic test results and medication prescriptions before and after the test. Results: A total of 450 patients (12.1 ± 4.3 years) diagnosed with anxiety disorder, attention deficit hyperactivity disorder, developmental disorders including autism, and/or a mood disorder received testing, and 435 of them were prescribed medications. Comparing data before and after testing, the total number of psychotropic prescriptions were reduced by 27.2% and the number of prescribed medications with severe gene-drug interactions decreased from 165 to 95 (11.4% to 8.9% of total medications prescribed). Approximately 40% of actionable genetic annotation were related to *CYP2CD6* and *CYP2C19*. Patients of Asian descent had significantly higher likelihood than other races of being classified as poor to intermediate metabolizers of antidepressants, mood stabilizers, and antipsychotics (*p* = 0.008, 0.007, and 0.001, respectively). Diagnoses, including autism spectrum disorder, were not associated with increased risks of severe gene-drug interactions. Conclusions: Pharmacogenetic testing in child and adolescent psychiatry is currently based on few clinically actionable genes validated by CPIC and/or FDA. Although this approach can be moderately utilized to guide psychotropic medication prescribing for pediatric patients with psychiatric disorders, clinicians should cautiously interpret test results while still relying on clinical experience and judgment to direct the final selection of medication.

## 1. Introduction

Approximately 16.5% (7.7 million) of youth aged 6- to 17-years old in the United States are diagnosed with at least one mental health disorder [1]. Among those aged 2 to 17 years, 9.4% have Attention Deficit Hyperactivity Disorder (ADHD) [2], 7.1% have anxiety disorder, and 3.2% have major depressive disorder [3]. These disorders are associated with significant morbidity [4]. As their prevalence increases, clinicians are challenged to find effective early treatments to avert disease progression. While an armamentarium of psychotropic medications is available, heterogeneity exists with treatment response and medication tolerance as attributed to factors such as gender, accuracy of diagnosis, and comorbidities. Additionally, genetics accounts for about 40% of the variability in antidepressant response within major depression [5]. As such, pharmacogenetics, which studies the impact of genetic variations on drug responses, is an area of increasing interest among clinicians seeking to incorporate genetics to personalize psychotropic treatments and to reduce the trial-and-error approach to prescribing.

Conflicting evidence exists regarding the clinical utility of pharmacogenetic testing in psychiatry despite a strong interest in adopting this approach to guide medication prescribing for adults with mental health disorders [6,7,8,9,10]. The evidence for pharmacogenetic-guided treatment of pediatric mental health disorders is more limited [11,12,13]. Further, while the Clinical Pharmacogenetics Implementation Consortium (CPIC) has pharmacogenetic guidelines for *CYP2D6* and atomoxetine, which are specifically based on pediatric studies, they added a cautionary statement for applying guidelines for other medications (e.g., SSRIs) in pediatric patients since most of the data were derived from adult study populations [14]. The ontologic impact of the expression of *CYP2D6* and *CYP2C19* drug metabolizing enzyme activity also adds another level of complexity that clinicians need to consider when applying pharmacogenetic results [15]. Despite this, the use of pharmacogenetic testing to guide treatment decision in pediatric psychiatric disorders is gaining traction [16,17,18], as more pediatric hospitals in the United States are adopting the test with some places testing specific genes while others use panel-based test [12,19,20,21,22]. 

While prior studies have evaluated panel-based pharmacogenetic testing in adults with mental health disorders, to our knowledge, there is little published literature on its use in children and adolescents with mental health disorders [18,23,24,25,26,27,28]. This study seeks to address this knowledge gap by evaluating the application of such testing among pediatric patients with neuropsychiatric disorders treated at a tertiary care hospital through a retrospective chart review. Demographic and diagnostic factors were examined to see if certain patient types are at higher risk to have severe gene-drug interactions, potentially suggesting which groups may benefit most from pharmacogenetic testing. Findings were described as trends and experiences in applying commercial pharmacogenetic testing among pediatric patients with neuropsychiatric disorders.

## 2. Materials and Methods

### 2.1. Study Design and Study Population

This is a retrospective analysis of pediatric patients receiving care for a psychiatric disorder in a large urban academic children’s hospital. Patients were seen in outpatient specialty clinics in neurology, developmental/behavioral pediatrics, adolescent medicine, and psychiatry, as well as in inpatient psychiatry units; all of which are operating within the same institution. During the study period, clinicians had started to use Genesight^®^ testing for patients who were receiving polypharmacy, experiencing medication side effects, or were not responding to current medication regimen. Inclusion criteria were patients aged 1 to 22 years (maximum age of patients seen in specialty clinics), with a diagnosis of psychiatric disorder, who had undergone psychiatric pharmacogenetic testing using GeneSight^®^ Psychotropic (Myriad Neuroscience) between January 2015 to November 2016. Pharmacogenomic test reports of patients meeting the inclusion criteria were obtained from Myriad Neuroscience who housed the data. This dataset was then merged with clinical dataset (e.g., basic demographic information, psychotropic medication prescribed any time before and after the pharmacogenomic test date until the end of the study period) that was extracted from the study site’s electronic medical database. All patient identifiers were removed after both pharmacogenomic and clinical datasets were merged, with the data managed using the Research Electronic Data Capture (REDCap) [29]. All data analyses were completed using the deidentified dataset.

### 2.2. Pharmacogenetic Testing

GeneSight^®^ Psychotropic test from Myriad Neuroscience (formerly Assurex Health, Inc., Mason, OH) tested 59 alleles and variants across 8 genes (*CYP1A2, CYP2C9, CYP2C19, CYP3A4, CYP2B6, CYP2D6, HTR2A, SLC6A4*) as reported elsewhere [30]. A proprietary algorithm that weighted the combined influence of a patient’s genotype results was applied to provide pharmacogenetic recommendations for 38 psychotropic medications based on three levels of gene-drug interaction: (i) ‘use as directed’ (no gene-drug interaction detected; annotated by green label in the test report); (ii) ‘use with caution’ (moderate gene-drug interaction and drug may be effective with dose modification; yellow label); and (iii) ‘use with increased caution and with more frequent monitoring’ (severe gene-drug interaction, which may significantly impact drug safety and/or efficacy; a red label) [30]. 

### 2.3. Prescription Trends Analysis 

Psychotropic medications prescribed in this study were categorized into antidepressants, antipsychotics, medications to treat ADHD, and mood stabilizers. These medications were analyzed for pharmacogenetic annotations based on CPIC guidelines [14] and the Food and Drug Administration (FDA) list of drugs with pharmacogenomic biomarkers in the drug label [31]. Psychotropic medications prescribed before and after the pharmacogenetic test within the study period were compared against the Genesight^®^ report to determine the type of gene-drug interaction. The study then focused on analyzing antidepressants to illustrate how pharmacogenetic results were applied clinically. Specifically, an analysis was performed on the selective serotonin reuptake inhibitors (SSRI; citalopram, escitalopram, fluvoxamine, paroxetine, fluoxetine and sertraline), serotonin norepinephrine reuptake inhibitors (SNRI; desvenlafaxine, duloxetine, levomilnacipran, venlafaxine), and dopamine norepinephrine reuptake inhibitor (DNRI; bupropion). Tricyclic antidepressants were excluded as they were less frequently prescribed clinically given their side effect profile and low utilization for depression in pediatric patients.

### 2.4. Statistical Analysis

De-identified dataset was analyzed using descriptive statistics (mean, standard deviation, frequency as count and percentage). The number and type of psychotropic medications prescribed for each subject before and after the pharmacogenetic test (within the study period) were compared. Correlational statistics (chi-square) was applied to determine whether pertinent predictors (age, gender, race, and psychiatric diagnoses) were related to patients receiving prescription for medications in the severe gene-drug interaction category. All analyses were performed using JMP^®^ Pro (version 14.1.0), and significance level was set at *p* < 0.05.

## 3. Results

### 3.1. Demographic and Medical Profile of the Study Cohort

A total of 28 clinicians tested 450 patients during the study period. These clinicians consisted of 12 child and adolescent psychiatrists, 7 child and adolescent psychiatry fellows, 3 pediatricians specialized in adolescent medicine, 3 developmental pediatricians, and 3 pediatric neurologists. The study cohort (N = 450) was 64% male, 76% Caucasian, with a mean age (SD) of 12 (4.3) years. The most common mental health diagnosis was an anxiety disorder (44%), followed by ADHD (38%), and autism spectrum disorder (ASD) (35%). Major depression was present in 15% of the cohort. Other depressive disorders such as disruptive mood dysregulation disorder, persistent depressive disorder (dysthymia), substance/medication-induced depressive disorder, depressive disorder due to another medical condition, other specified depressive disorder, and/or unspecified depressive disorder were present in 25% of the cohort. The majority of the patients (42%) had two concurrent psychiatric diagnoses, and 18% had at least three concurrent psychiatric diagnoses. All diagnoses were based on DSM–5 criteria [32]. Table 1 lists the basic demographic and medical profile of the study cohort.

### 3.2. Prescription Trends of Psychotropic Medications 

Among the 450 patients who underwent psychiatric pharmacogenetic testing, only 435 (97%) were prescribed psychotropic medications. A total of 47 different psychotropic medications were prescribed to these patients during the study period (Table 2). Most of the medications were antidepressants (40%), followed by antipsychotics (28%), ADHD medications (17%), and mood stabilizers (15%). The top 10 psychotropic medications prescribed included guanfacine (50%), sertraline (43%), risperidone (35%), methylphenidate (33%), aripiprazole (33%), fluoxetine (29%), mixed amphetamine salts (25%), lamotrigine (19%), atomoxetine (16%), and lisdexamfetamine (15%) (Table 2). Data collected from the time before the pharmacogenetic test was conducted showed that there were 1469 prescriptions written for these patients (an average of 3.3 prescriptions per patient). Data from time period after the test indicated that there were 1070 prescriptions (an average of 2.5 prescriptions per patient). Overall, this reflected a decrease of 27.2% in the number of prescriptions. Additionally, the number of prescribed medications with severe gene-drug interactions (as rated by GeneSight**^®^** algorithm) decreased from 165 prior to testing to 95 following the testing (i.e., from 11.4% to 8.9% of total medications prescribed).

### 3.3. Predictors of Severe Gene-Drug Interactions

Based on their pharmacogenetic test results, Asians had significantly more severe gene-drug interactions for antidepressants and mood stabilizers than patients of other races (*p* = 0.008 and *p* = 0.007, respectively). A similar trend was observed when comparing Asians with Caucasians for antipsychotics (*p* = 0.001). However, age, gender, and psychiatric diagnoses were not significant predictors of which patients would be at risk for severe gene-drug interactions (*p* > 0.05 for all comparisons). Notably, a diagnosis of ASD did not predict a higher likelihood of severe gene-drug interactions.

### 3.4. Psychotropic Medications with Pharmacogenetic Annotation

**Figure 1:**
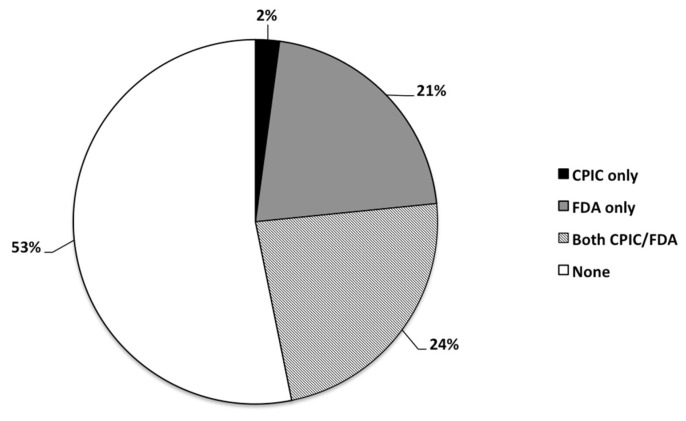
Frequency distribution (as percentage) of psychotropic medications (N = 47) being prescribed to the study cohort (N = 435) having CPIC and/or FDA guidelines, or none at all.

Approximately half of the medications listed in the GeneSight**^®^** algorithm (53%) did not have any pharmacogenetic annotation from CPIC or the FDA; while 11 (24%) had information from CPIC and FDA, 10 (21%) had information from FDA alone, and 1 (2%) had information from CPIC alone (N = 47; Figure 1). 

The majority of the pharmacogenetic annotations were related to *CYP2CD6* and *CYP2C19* (40%), followed by HLA-A and HLA-B genes for drug hypersensitivity reaction (4%) and POLG gene, which predicted the risk for valproate-induced hepatotoxicity (2%) (Table 2). Since *CYP2D6* and *CYP2C19* were the most frequently noted metabolizer phenotypes, the rates of these phenotypes of the study cohort (as derived from the Genesight^®^ reports) were compared against trends of general population published in literature [33,34]. Only trends for Caucasians were analyzed due to sample size. This comparative analysis was performed to evaluate whether *CYP2D6* and *CYP2C19* metabolizer frequency distribution of our study cohort was comparable to the general population, considering that the patients in our cohort were potentially more likely to have comorbidities and treatment failures since they were seen at a tertiary care center. When compared to data from published literature [33,34], Caucasians in this study cohort had similar *CYP2D6* and *CYP2C19* metabolizer frequency distribution as the general population. For *CYP2D6* metabolizer phenotype, the majority of the study cohort were normal metabolizers (80.3%; compared to 80.9% in general population), followed by intermediate (9.7% vs. 7.8%), poor (7.7% vs. 6.6%), ultrarapid (2.4% vs. 3.5%), and normal (0% vs. 1.2%). For *CYP2C19* metabolizer phenotype, the study cohort was mostly of normal metabolizer phenotype (40.1%; compared to 42.0% in general population), followed by rapid (27.0% vs. 27.0%), intermediate (26.7% vs. 19.0%), ultrarapid (4.8% vs. 4.2%), and poor (1.5% vs. 2.8%).

### 3.5. Pharmacogenetic Results for Antidepressants (SSRIs, SNRIs, DNRIs)

**Figure 2:**
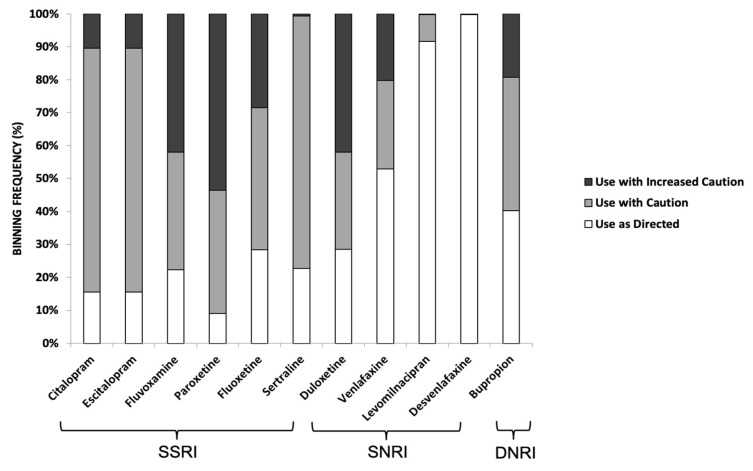
Binning frequency distribution of SSRI, SNRI, and DNRI, which were prescribed to the study cohort based on their GeneSight**^®^** pharmacogenetics reports. Color codes for respective bins were based on the classification outlined by Jablonski et al. [30]. These are: (i) ‘use as directed’ (no gene-drug interaction detected; annotated by green label in the test report); (ii) ‘use with caution’ (moderate gene-drug interaction and drug may be effective with dose modification; annotated by a yellow label); and (iii) ‘use with increased caution and with more frequent monitoring’ (severe gene-drug interaction that may significantly impact drug safety and/or efficacy; as annotated by a red label).

Among the SSRIs, paroxetine (54%) had the highest proportion of patients with severe gene-drug interactions (red label) followed by fluvoxamine (42%), then fluoxetine (29%). Among the SNRIs, duloxetine (42%) had the largest proportion of patients with severe gene-drug interactions followed by venlafaxine (20%). In contrast, desvenlafaxine (an SNRI) had no gene-drug interaction (green label). For bupropion (a DNRI), 19% of the study cohort had severe gene-drug interaction. These results are summarized in Figure 2.

## 4. Discussion

Increasing prevalence of mental health disorders among youth has created a relative shortage of pediatric specialists (e.g., child and adolescent psychiatrists, pediatric neurologists, developmental and behavioral pediatricians, among others) to provide evaluation and treatment. Commercial panel-based pharmacogenetic testing has been introduced to the field of psychiatry as a way to guide prescribing practices. Currently, the use of pharmacogenomic testing prior to prescribing psychotropic medications in pediatric patients is not mandatory, nor it is a routine practice. Such testing is typically performed once a pediatric patient has had unanticipated medication side effects, has a history of failed medications, or is initiating a medication for which there is a clearly defined guideline response (e.g., carbamazepine and HLA-B*1502). While its role in child and adolescent psychiatry continues to be defined, many clinicians have adopted such testing clinically, or find their patients’ caregivers bringing in these results for them to interpret. Since there are limited data in this field based on pediatric populations, this retrospective study primarily sought to describe trends observed from a large cohort of pediatric patients with psychiatric disorders seen at a tertiary care center who had undergone such testing. A secondary outcome to this study was to highlight pertinent points garnered from our observation that clinicians could consider when using commercial pharmacogenetic testing to guide prescribing practices in light of current evidence.

There are currently more than 200 medications with pharmacogenetic biomarker information in FDA-approved labels, with 39 of them being prescribed for psychiatric conditions [31,35]. Among psychotropic medications, the main biomarkers involved are the hepatic cytochrome P450 2C19 and 2D6 metabolizing enzymes [36]. CPIC has also published evidence-based peer-reviewed pharmacogenetic guidelines for tricyclic antidepressants and SSRIs based on *CYP2D6* and *CYP2C19* genotypes, and atomoxetine based on *CYP2D6* genotypes [14,37,38]. The interest to personalize medication prescribing in psychiatry has fueled the development of commercial pharmacogenetic tests with several marketed as being psychiatry focused [39]. Many of these tests, however, included other genes of varying pharmacogenetic level of evidence in addition to *CYP2D6* and *CYP2C19.* Patients’ test results may be reported in the form of a pharmacogenetic decision support tool, such as classifying medications in color-coded bins based on the level of gene-drug interaction. However, these categorizations are based on proprietary algorithms that are not standardized across the different companies [39,40,41]. Clinicians need to be aware of these points when selecting a pharmacogenetic test for their patients or when being presented with commercial test reports for clinical interpretation. 

Anxiety and mood/depressive disorders are common diagnoses in our study cohort. It has been estimated that more than half of all adult patients with major depressive disorders fail to achieve remission with the initial antidepressant regimen, with the pediatric literature reporting a wider variability (23 to 63%) [42]. This factor has driven the interest for incorporating pharmacogenetics to guide medication selection, especially to aid in identifying medications with a low side effect profile or adjusting dosage to reflect a patient’s metabolic phenotype. While CPIC guidelines provide recommendations on drug selection and dosing based on *CYP2C19* and *CYP2D6* genetic polymorphisms, which alter the metabolism of certain antidepressants, other pharmacodynamic factors remain to be elucidated since the biology of mental health disorders remains to be fully understood [43,44]. Clinicians who utilize pharmacogenetic tests should also be cognizant of CPIC and FDA guidelines, especially since CPIC recommendations for the pediatric population were formulated based on current pharmacogenetic evidence and continues to be updated [14,31]. Further, a systematic review of drugs with pharmacogenetic information in FDA drug labels found 65 drugs with pharmacogenetic information that have been evaluated in children, but a majority (86%) of the pharmacogenetic information were extrapolated from adult studies [45]. As such, clinicians need to moderate the expectations of patients and their caregivers toward results of pharmacogenetic testing by explaining the role and limitations of these tests, especially when applying adult-derived pharmacogenetic information towards the care of their pediatric patients.

Another practical consideration is related to the clinical interpretation of pharmacogenetic test results for genetic variants that have mixed evidence. The FDA issued a warning statement expressing concerns over pharmacogenetic tests that claim to “predict a patient’s response to specific medications that have not been reviewed by the FDA and may not be supported by clinical evidence” [46]. As a case example, a certain genetic test claimed that their test could identify the extent of efficacy of select antidepressants when compared to others. This is troubling because the relationship between DNA variations and physiological processes influencing antidepressant efficacy has not been established [46]. The FDA also expressed worries that certain software programs that interpreted genetic information made similar claims when current evidence does not support those claims [47]. Reflecting on these cases, the FDA was concerned that clinicians who largely relied on the results of pharmacogenetic tests might make inappropriate medication changes that could inadvertently result in adverse health consequences. Consequently, the FDA has reached out to several pharmacogenetic testing companies who have since removed specific medication names from their promotional material and patient test reports. The FDA also reiterated their commitment to supporting innovation in this area given the evidence available for some gene-drug pairs. While the FDA continues their monitoring efforts, clinicians can consult resources such as FDA-approved drug labels and CPIC guidelines on how to use genetic information to guide their prescribing [14,31].

With regards to the role of pharmacogenetic testing in psychiatry medicine, the International Society of Psychiatry Genetics (ISPG) issued a genetic testing statement that “Pharmacogenetic testing should be viewed as a decision-support tool to assist in thoughtful implementation of good clinical care, enhancing rather than offering an alternative to standard protocols” [48]. While genetics is one piece of information that can be used to guide medication prescribing, other clinical factors (e.g., renal function, and medical and psychiatric comorbidities) must also be considered. The analysis of the antidepressants (SSRIs, SNRIs, and DNRIs) prescribed in our genotyped pediatric cohort showed that a majority of their pharmacogenetic recommendations were in the moderate-to-severe gene-drug interaction category (yellow or red designation in the test reports) (Figure 2), which may alarm some clinicians (and their patients and caregivers). However, clinically in many cases, such classification does not necessarily lead to contraindications that require medication change. Instead, there are other strategies that can be implemented including dosing adjustment and closer monitoring of side effects. Clinicians at the study site may have used these strategies since we observed a reduction in polypharmacy in our study cohort post-testing (M.Z.W.; through personal communications and case discussions). Thus, clinical insights are still necessary to guide prescribing practices instead of completely relying on pharmacogenetic test results.

Many clinicians believe commercial pharmacogenetic testing has moderate clinical utility, despite its limitations and conflicting evidence in the literature [49]. However, these findings may be skewed in favor of pharmacogenetic testing since several of these studies were performed at sites where clinicians were already familiar with pharmacogenetics [50,51,52,53]. A recent survey of clinicians also indicated that 85% of respondents were concerned about the lack of clear guidance for clinical application [54]. This trend is troubling considering that many clinicians who use this type of test to guide clinical decision-making may not have an extensive pharmacogenetic knowledge to be sufficiently proficient in interpreting the test reports, among other perceived barriers [20,49,55]. Considering these factors, a multidisciplinary approach should be considered when interpreting pharmacogenetic test results. Psychiatric pharmacists trained in pharmacogenetics can provide additional clinical support in this way. However, a survey of psychiatric pharmacists indicated that only 36% considered themselves to be knowledgeable in pharmacogenetics [52]. Medical liaisons from pharmacogenetic testing vendors could provide another support resource, although their expertise may not translate to medical realm. Overall, this highlights the need of providing more pharmacogenetic training and other resources for healthcare providers, which is consistent with the findings of Liko et al. [49]. 

Logistical variables related to ordering the pharmacogenetic test (e.g., where to obtain the test, on whom and when to perform the test, costs to patients, and other pertinent factors) should be discussed at the clinic visit. A recent survey of psychiatrists noted that 94% of respondents were concerned about the cost associated with pharmacogenetic testing [54], which may not be covered by insurance providers. The cost of pharmacogenetic testing may be a barrier to its uptake, as most patients currently pay out-of-pocket for the test. In the United States, many Medicare contractors do not consider the test reasonable or necessary, and private insurers tend to follow Medicare coverage decisions [56]. However, this is gradually changing [57]. In 2019, United Healthcare agreed to reimburse pharmacogenetic panel-based testing, as it is proven and medically necessary to guide antidepressant and antipsychotic prescribing under certain circumstances [58]. Additionally, Medicare expanded their coverage through new Molecular Diagnostic Services (MolDx) local coverage determinations starting in 2020 onwards. A cost-effectiveness study comparing pharmacogenetic guided strategy versus treatment-as-usual in guiding antidepressant treatment in Canadian patients showed that the pharmacogenetic guided strategy was more efficacious and less costly compared to the latter [59]. It should be noted that pediatric data are lacking since most cost-benefit studies on pharmacogenetic testing in psychiatry have been conducted in adult populations. The majority of the pharmacoeconomic evaluation studies are also based on simulations rather than actual clinical data [60]. Therefore, future studies can be conducted to evaluate the cost-benefit of using pharmacogenetic testing to guide drug treatment in pediatric patients with neuropsychiatric disorders, and address payer concerns regarding its clinical utility and benefits. 

Meanwhile, clinicians might consider ordering pharmacogenetic test for select patients, such as those with history of psychotropic medication intolerance, and/or repeated treatment failure. Our results indicated that patients of Asian descent were more likely to have significant gene-drug interactions, hence those patients may be candidates for such testing as well. Overall, it is imperative that clinicians communicate these logistical variables, as well as the potential utility and limitations of pharmacogenetic testing when discussing testing plan and subsequent results with their patients and caregivers.

There are several limitations to this study. First, the study did not set out to determine the clinical impact of prescribing decisions as a result of pharmacogenetic testing, but rather provided a brief description of prescribing trends of various psychotropic medications based on the pharmacogenetic results of the study cohort. The retrospective study design that was utilized in this study is certainly not the gold standard for objectively measuring symptomatology and long-term impact of prescribed medications. Our interpretation about the impact of pharmacogenetic testing at the study site was also influenced by the variability in the detailed information recorded in patients’ charts. While the study cohort’s medical record did not systematically document reasons for medication changes, most of the clinicians ordered the pharmacogenetic test when the initial medication regimens were either not effective and/or were causing significant side effects to the patient (M.Z.W.; personal communications and clinical case discussions). Further, the study was conducted in a single tertiary care center and thus the results may not be generalizable to other pediatric medical centers due to differing practice preferences and hospital formularies. This study also included a diverse study population (i.e., patients from both inpatient and outpatient settings), which may have introduced confounders. Despite these limitations, the findings remain valuable to the literature as it reflects a snapshot of ‘real world’ practice where pediatric patients were selected for genotyping based on clinical consideration.

## 5. Conclusions

In summary, commercial pharmacogenetic tests can be utilized to guide psychotropic medication prescribing for pediatric patients with psychiatric disorders while recognizing their limitations. In the context of pediatric pharmacogenetic testing, clinicians need to be aware of the ontologic effects of CYP450 drug metabolizing enzyme development in children. Clinicians should also use pharmacogenetic resources such as the CPIC and/or FDA guidelines to corroborate gene-drug(s) pairs that have strong evidence to support their use in the pediatric cohort. We anticipate that the debate surrounding the clinical utility of pharmacogenetic test for pediatric patients with psychiatric disorders continues as long as large randomized pharmacogenetic studies based on pediatric cohort remain unexplored, and that clinicians are not adequately trained in pharmacogenetics to effectively interpret test results. Ultimately, the results of commercial panel-based pharmacogenetic testing should not replace clinical experience and judgment for making the final medication choice.

## Figures and Tables

**Table 1:** Basic demographic and medical profiles of study cohort (N = 450).

Characteristics	Result
Age (in years)—mean (SD)	12 (4.3)
Male—n (%)	289 (64)
Race—n (%)	
White/Caucasian	340 (76)
Other	33 (7)
Multi-racial	29 (6)
Unknown/Not reported	24 (5)
Black/African American	14 (3)
Asian	10 (2)
Ethnicity—n (%)	
Not Hispanic or Latino	362 (81)
Hispanic or Latino	54 (12)
Unknown/Not reported	34 (8)
Mental health diagnosis—n (%)	
Anxiety Disorder	199 (44)
ADHD ^a^	172 (38)
ASD ^a^	156 (35)
Any Mood Disorder	144 (32)
Other Depressive Disorder	114 (25)
MDD ^a^	67 (15)
No. of patients with concurrent psychiatric diagnoses—n (%)	
1 diagnosis	176 (39)
2 diagnoses	189 (42)
3 diagnoses	81 (18)
≥4 diagnoses	4 (0.9)

^a^ Abbreviations: Attention Deficit Hyperactivity Disorder (ADHD), Autism Spectrum Disorder (ASD), Major Depressive Disorder (MDD).

**Table 2:** Frequency distribution of psychotropic medications (N = 47; as percentage; in descending order of prescribing frequency) being prescribed to the study cohort (N = 435) at any time during the study period, and their respective pharmacogenetics information.

Generic Name	Medication Type	Prescribing Frequency (%)	Guideline	Genotype
Guanfacine	ADHD Medication	49.9	None	None
Sertraline	Antidepressant	43.0	CPIC	*CYP2C19*
Risperidone	Antipsychotic	34.9	FDA	*CYP2D6*
Methylphenidate	ADHD Medication	33.3	None	None
Aripiprazole	Antipsychotic	32.6	FDA	*CYP2D6*
Fluoxetine	Antidepressant	29.4	FDA	*CYP2D6*
Mixed amphetamine salts	ADHD Medication	25.3	None	None
Lamotrigine	Mood stabilizer	18.9	None	None
Atomoxetine	ADHD Medication	16.3	CPIC, FDA	*CYP2D6*
Lisdexamfetamine	ADHD Medication	14.9	None	None
Citalopram	Antidepressant	14.7	CPIC, FDA	*CYP2C19, CYP2D6*
Trazodone	Antidepressant	14.7	None	None
Quetiapine	Antipsychotic	14.5	None	None
Dexmethylphenidate	ADHD Medication	13.3	None	None
Escitalopram	Antidepressant	12.6	CPIC, FDA	*CYP2C19, CYP2D6*
Valproic acid	Mood stabilizer	9.0	FDA	*POLG*
Olanzapine	Antipsychotic	8.3	None	None
Topiramate	Mood stabilizer	8.3	None	None
Venlafaxine	Antidepressant	8.3	FDA	*CYP2D6*
Bupropion	Antidepressant	7.6	None	None
Desvenlafaxine	Antidepressant	7.4	FDA	*CYP2D6*
Oxcarbazepine	Mood stabilizer	7.4	CPIC, FDA	HLA-B*1502
Gabapentin	Mood stabilizer	6.9	None	None
Clonidine	ADHD Medication	6.5	None	None
Lithium	Mood stabilizer	6.4	None	None
Amitriptyline	Antidepressant	5.5	CPIC, FDA	*CYP2C19, CYP2D6*
Lurasidone	Antipsychotic	4.1	None	None
Ziprasidone	Antipsychotic	3.7	None	None
Duloxetine	Antidepressant	2.8	FDA	*CYP2D6*
Fluvoxamine	Antidepressant	2.5	CPIC, FDA	*CYP2D6*
Dextroamphetamine	ADHD Medication	2.1	None	None
Mirtazapine	Antidepressant	1.8	None	None
Asenapine	Antipsychotic	1.6	None	None
Carbamazepine	Mood stabilizer	1.2	CPIC, FDA	HLA-B*1502, HLA-A*3101
Vilazodone	Antidepressant	0.9	None	None
Clomipramine	Antidepressant	0.7	CPIC, FDA	*CYP2D6*
Paliperidone	Antipsychotic	0.7	None	None
Clozapine	Antipsychotic	0.5	FDA	*CYP2D6*
Imipramine	Antidepressant	0.5	CPIC, FDA	*CYP2D6, CYP2C19*
Doxepin	Antidepressant	0.2	CPIC, FDA	*CYP2C19, CYP2D6*
Haloperidol	Antipsychotic	0.2	None	None
Iloperidone	Antipsychotic	0.2	FDA	*CYP2D6*
Levomilnacipran	Antidepressant	0.2	None	None
Paroxetine	Antidepressant	0.2	CPIC, FDA	*CYP2D6*
Perphenazine	Antipsychotic	0.2	FDA	*CYP2D6*
Selegiline	Antidepressant	0.2	None	None
Thiothixene	Antipsychotic	0.2	None	None
